# Interference of Quercetin on Astragalus Polysaccharide-Induced Macrophage Activation

**DOI:** 10.3390/molecules23071563

**Published:** 2018-06-28

**Authors:** Zhi-Peng Li, Hong-Bing Liu, Quan-Wei Zhang, Li-Feng Li, Wan-Rong Bao, Dik-Lung Ma, Chung-Hang Leung, Zhao-Xiang Bian, Ai-Ping Lu, Quan-Bin Han

**Affiliations:** 1 School of Chinese Medicine, Hong Kong Baptist University, Hong Kong, China; lizhipeng0903@163.com (Z.-P.L.); LHB51219031@gmail.com (H.-B.L.); elvinzhang@hkbu.edu.hk (Q.-W.Z.); 16483294@life.hkbu.edu.hk (L.-F.L.); 16483502@life.hkbu.edu.hk (W.-R.B.); bzxiang@hkbu.edu.hk (Z.-X.B.); 2 Department of Chemistry, Hong Kong Baptist University, Kowloon Tong, Hong Kong, China; edmondma@hkbu.edu.hk; 3 State Key Laboratory of Quality Research in Chinese Medicine, Institute of Chinese Medical Sciences, University of Macau, Macao, China; duncanleung@umac.mo

**Keywords:** astragalus polysaccharide, quercetin, cytokines, macrophage, foods

## Abstract

Polysaccharides, which exert immunoregulatory effects, are becoming more and more popular as food supplements; however, certain components of ordinary foods could be reducing the polysaccharides beneficial effects. Quercetin, a flavonoid found in common fruits and vegetables, is one such component. This study investigated the effects of quercetin on Astragalus polysaccharide RAP induced-macrophage activation. The results show quercetin decreases the NO production and iNOS gene expression in RAW264.7 cells, and it inhibits the production of cytokines in RAW264.7 cells and peritoneal macrophages. Western blot analysis results suggest that quercetin inhibits the phosphorylation of Akt/mTORC1, MAPKs, and TBK1, but has no effect on NF-κB in RAP-induced RAW264.7 cells. Taken together, the results show that quercetin partly inhibits macrophage activation by the Astragalus polysaccharide RAP. This study demonstrates that quercetin-containing foods may interfere with the immune-enhancing effects of Astragalus polysaccharide RAP to a certain extent.

## 1. Introduction

Many vegetables and fruits that contain flavonoids with anti-inflammatory properties are consumed daily [1,2,3]. Quercetin is a potent bioactive flavonoid with various beneficial effects on human health. It is a typical dietary flavonoid in a wide range of foods, including fruits, vegetables, tea, nuts, and seeds [4]. Studies have revealed that quercetin has many biological functions [5,6,7]. It can promote free radical scavenging and exert strong antioxidant effects [8,9,10]. Recent studies have demonstrated that quercetin has anti-inflammatory effects [11,12] and immunosuppressive effects [13,14]. As for the mechanism, it has been reported that quercetin inhibits Nuclear factor-κB (NK-κB), extracellular signal-regulated kinase (ERK) 1/2 and c-Jun N-terminal kinase (JNK) activation in Lipopolysaccharides (LPS)-induced inflammatory response [15]. It has also been reported that quercetin exerts its anti-inflammatory effects by suppressing the activation of ERK and p38 mitogen-activated protein kinase (p38 MAP kinase), and NF-κB signal transduction pathways; however, it does not suppress the activation of JNK kinase [11].

Polysaccharides as dietary supplements are now becoming popular. Many of these natural polysaccharides have been reported to have immunoenhancing effects [16,17]. In our preliminary study, a water soluble polysaccharide (RAP) was prepared from Radix Astragali and its structure was well characterized by a series of chemical and spectral analyses [18]. We demonstrated that RAP exerted its immunoenhancing effects on macrophage via the Toll-like receptor 4 (TLR4 receptor). Further study demonstrated that RAP could activate MAPKs and NF-κB downstream of the MyD88-dependent pathway, and it could activate TANK-binding kinase 1/interferon regulatory factor 3 (TBK1/IRF3) downstream of the MyD88-independent pathway [18].

The Astragalus polysaccharide RAP can enhance immune system activity, however, quercetin can exert immunosuppressive effects. Hence, the question arises as to whether eating foods with quercetin can compromise the effects of Astragalus polysaccharides at the same time. To begin to address this, we investigated the effect of quercetin on one of the known biochemical pathways by which Astragalus polysaccharide exerts its effects. Specifically, we investigated whether quercetin inhibits Astragalus polysaccharide RAP-induced activity in macrophage cells. This study provides evidence of the interaction between certain foods and Astragalus polysaccharides that is critical for people who want to derive the most benefit from Astragalus polysaccharide supplements.

## 2. Results

### 2.1. Quercetin Did Not Exhibit Cytotoxicity on RAW264.7 and Peritoneal Macrophages Cells

**Figure 1:**
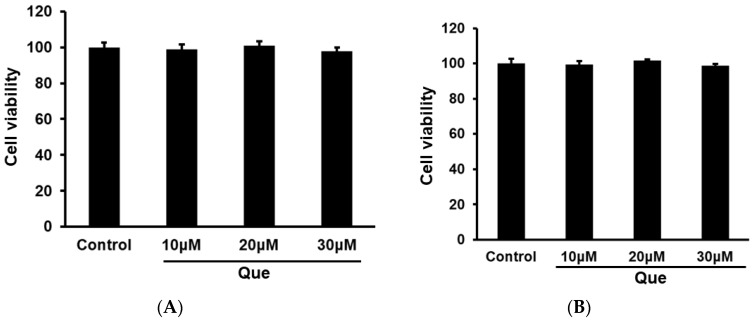
Quercetin did not exert cytotoxicity on RAW264.7 and peritoneal macrophages cells. RAW264.7 (**A**) and peritoneal macrophages cells (**B**) were incubated with Quercetin (10, 20 and 30 μM) for 24 h. The cell viability was examined by CCK8 assay.

Cell viability was examined to evaluate whether quercetin exhibited cytotoxicity on RAW264.7 and peritoneal macrophages cells. As shown in Figure 1, quercetin at three concentrations (10, 20, 30 μM) did not exert toxic effects on RAW264.7 and peritoneal macrophages cells.

### 2.2. Effect of Quercetin on Production of NO, TNF-α and IL-6 in RAP and LPS Induced RAW264.7 Cells

**Figure 2:**
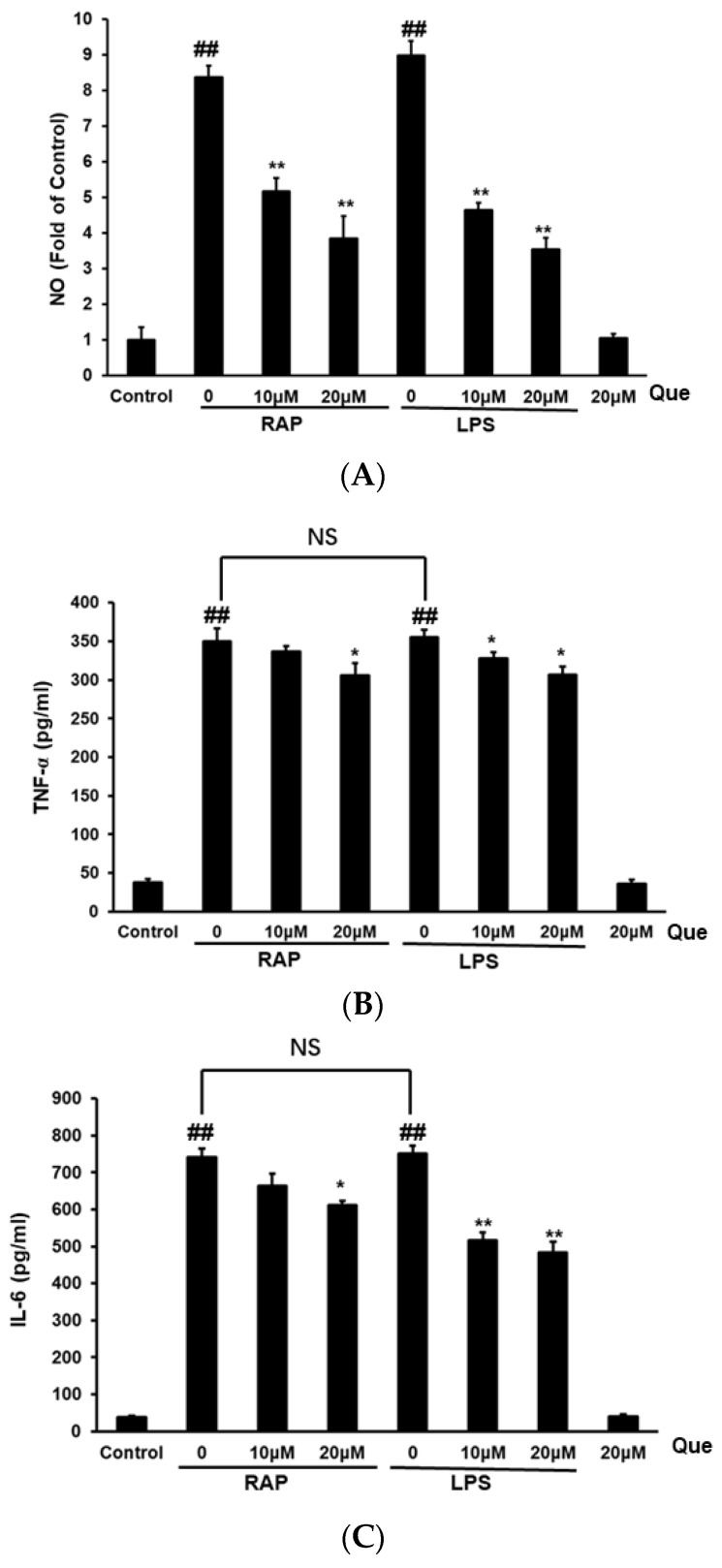
Effects of quercetin on nitric oxide (NO) (**A**), TNF-α (**B**) and IL-6 (**C**) production in RAP- and LPS-induced RAW264.7 macrophages. RAW264.7 cells were incubated with quercetin (10, 20 μM) and then treated with RAP (100 μg/mL) or LPS (100 ng/mL) for 24 h. Data from the experiments are expressed as mean ± SD (## *p* < 0.01 compared with control group; * *p* < 0.05, ** *p* < 0.01 compared with the RAP or LPS group).

As shown in Figure 2, RAP and LPS increased nitric oxide (NO) production from the basal level in RAW264.7 cells. The results show quercetin reduced NO production in RAW264.7 cells induced by LPS and RAP. The levels of tumor necrosis factor-alpha (TNF-α) and Interleukin-6 (IL-6) in RAW264.7 cells were examined by enzyme-linked immunosorbent assay (ELISA). After stimulation with LPS or RAP for 24 h, cells showed clearly increased levels of the cytokines TNF-α, and IL-6 as compared with control cells. The results showed that quercetin significantly reduced the production of TNF-α and IL-6 levels in RAW264.7 cells induced by LPS and RAP. The results demonstrate that quercetin inhibits macrophage activation by the Astragalus polysaccharide RAP, it has a similar effect with the inhibitory effect in LPS induced macrophage activation.

### 2.3. Effect of Quercetin on Gene Expression of iNOS, TNF-α, and IL-6 in RAP-Induced RAW264.7 Cells

**Figure 3:**
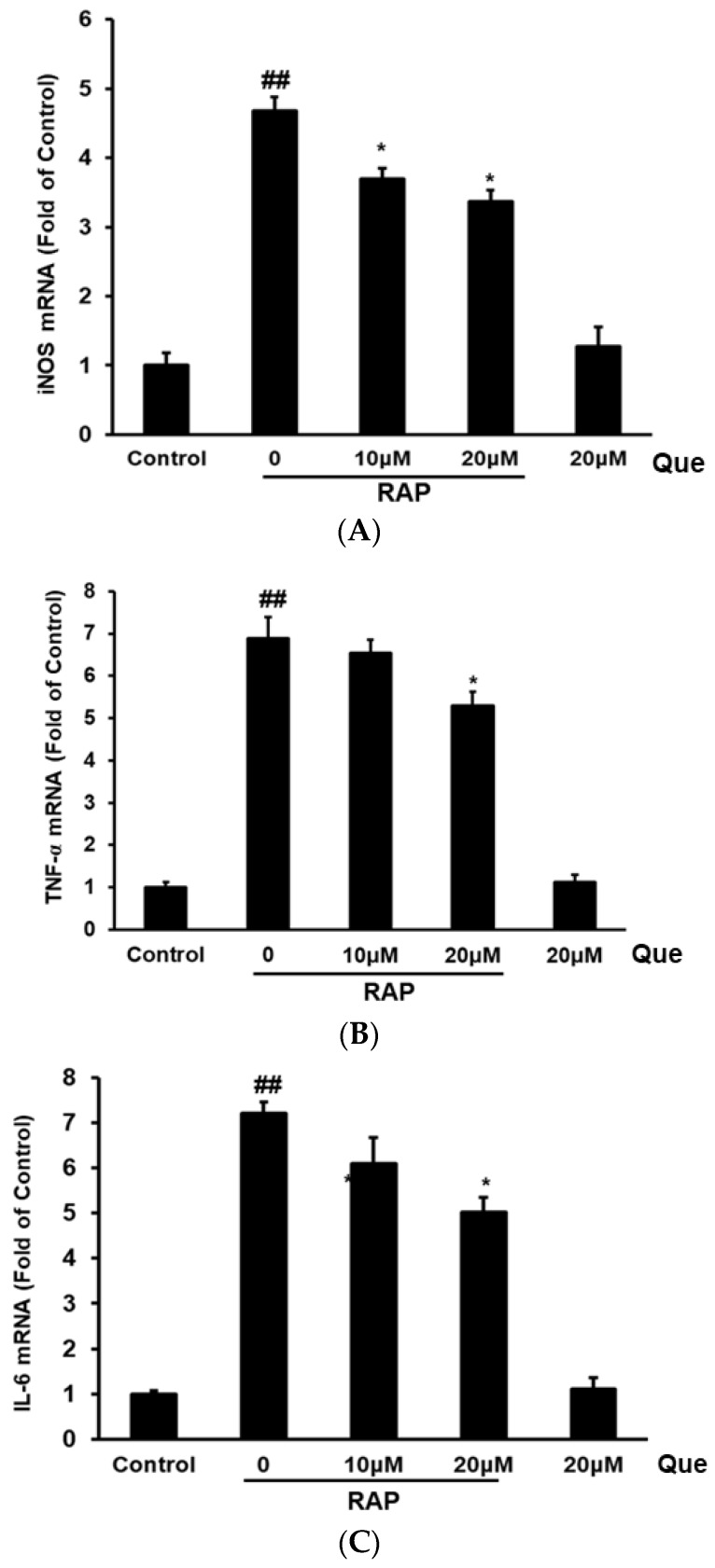
Effects of quercetin on inducible nitric oxide synthase (iNOS) (**A**), TNF-α (**B**) and IL-6 (**C**) mRNA expression in RAP-induced RAW264.7 macrophages. RAW264.7 cells were treated with quercetin (10, 20 μM) and then treated with RAP (100 μg/mL). The mRNA expression level was quantified with real time fluorescence PCR. Data from the experiments are expressed as mean ± SD (## *p* < 0.01 compared with control group; * *p* < 0.05, ** *p* < 0.01 compared with RAP group).

After examining the inflammatory factors production in the supernatant of the RAW264.7 cells, the gene expression of iNOS, TNF-α, and IL-6 were examined by RT-PCR. As shown in Figure 2, after RAP treatment, the mRNA expression of inducible nitric oxide synthase (iNOS), TNF-α, and IL-6 was strongly increased compared with control cells. The results show that quercetin significantly reduced iNOS gene expression in a dose-dependent manner, and, at high concentrations, quercetin reduced both TNF-α and IL-6 gene expression in RAP induced RAW264.7 cells (Figure 3).

### 2.4. Effect of Quercetin on Production of TNF-α and IL-6 in RAP-Induced Peritoneal Macrophages Cells

**Figure 4:**
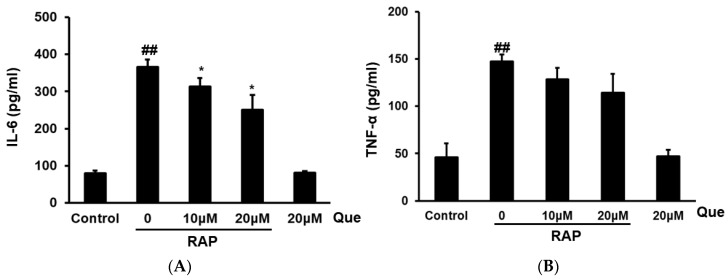
Effects of quercetin on IL-6 (**A**) and TNF-α (**B**) production in RAP-induced peritoneal macrophages. Peritoneal macrophages cells were incubated with quercetin (10, 20 μM) and then treated with RAP (100 μg/mL) for 24 h. Data from the experiments are expressed as mean ± SD (## *p* < 0.01 compared with control group; * *p* < 0.05, ** *p* < 0.01 compared with RAP group).

After investigating quercetin’s various inhibitory effects in RAW264.7 cells, we further examined the inhibitory effect of quercetin on inflammatory mediators in peritoneal macrophages. The production of TNF-α and IL-6 in peritoneal macrophages cells was tested by ELISA assay. After RAP stimulation for 24 h, the cytokines TNF-α, and IL-6 were strongly increased compared with the untreated control cells. The results showed that quercetin significantly reduced the production of IL-6 in a dose-dependent manner in peritoneal macrophages cells induced by RAP, however, quercetin exert only a slight inhibitory effect on the TNF-α level in peritoneal macrophages cells induced by RAP. The results were consistent with the results from RAW264.7 cells (see Figure 4).

### 2.5. Quercetin Inhibited the COX2 and iNOS in RAP-Induced RAW264.7 Cells

**Figure 5:**
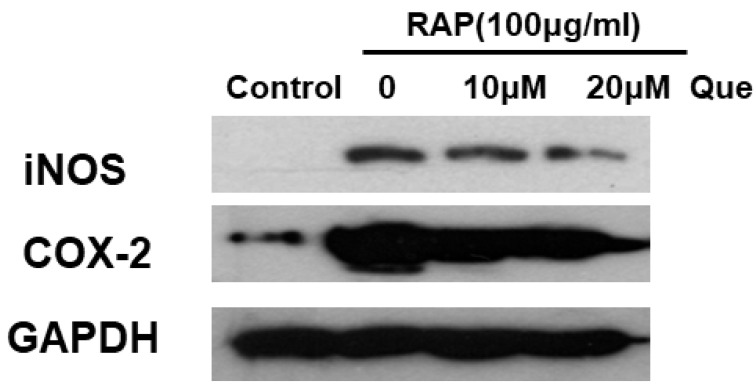
Effects of quercetin on RAP-induced COX2 and iNOS expression in RAW264.7 macrophages. RAW264.7 cells were treated with quercetin (10, 20 μM) for 2 h and then treated with RAP (100 μg/mL) for 24 h.

The inflammatory protein expression was assessed further after examining the production of inflammatory mediators. The inflammatory protein COX2 and iNOS were detected by Western blot analysis, and the results indicate that, as shown in Figure 5, quercetin significantly decreases the RAP-stimulated expression of COX2 and iNOS.

### 2.6. Effect of Quercetin on MyD88 Dependent and Independent Signaling Pathway in RAP-Induced RAW264.7 Cells

**Figure 6:**
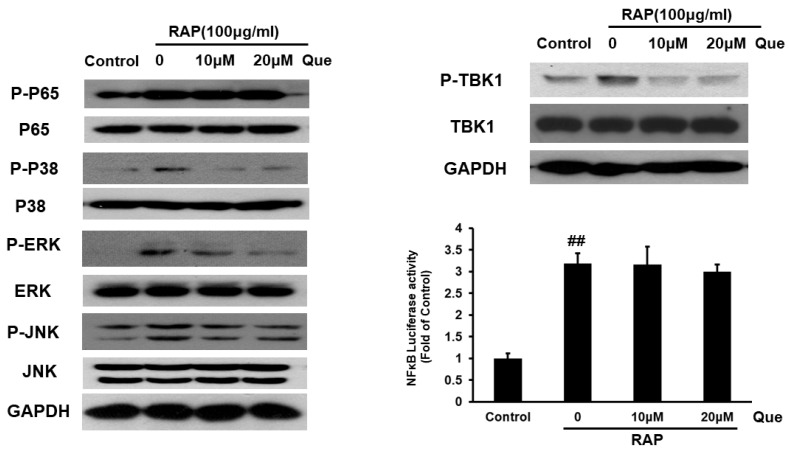
Effects of quercetin on RAP-induced p65, p38 MAPK, ERK1/2, JNK, TBK1 phosphorylation and NFκB luciferase activity in RAW264.7 macrophages. RAW264.7 cells were treated with quercetin (10, 20 μM) for 2 h and then treated with RAP (100 μg/mL) for 30 min. Protein samples were analyzed by western blot. 24 h after transfection, RAW264.7 cells were pretreated for 2 h with quercetin and then stimulated with RAP for 12 h. Then luciferase reporter activity was assayed (## *p* < 0.01 compared with control group).

The NF-κB and MAPKs pathways that are downstream of the MyD88 dependent pathway were examined. Western blot analysis indicated that quercetin can reduce the phosphorylation level of P38, ERK, and JNK, but has no effect on phosphorylation level of P65 and NF-κB activity in RAP-induced RAW264.7 cells. After examining the MyD88-dependent pathway, the TBK1 pathway downstream MyD88-independent pathway also should be detected, we measured the changes of TBK1 phosphorylation in RAW264.7 cells induced by RAP using Western blot analysis. The results showed that that treatment with quercetin can reduce TBK1 phosphorylation. The results demonstrate quercetin inhibits the phosphorylation of MAPKs and TBK1, but has no effect on NF-κB in RAP-induced RAW264.7 cells (see Figure 6).

### 2.7. Quercetin Inhibited Akt and mTORC1 in RAP-Induced RAW264.7 Cells

**Figure 7:**
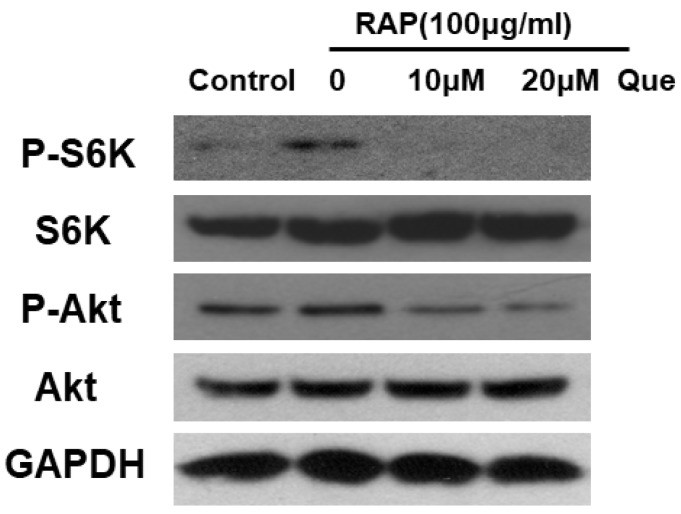
Activation of Akt and mTORC1/S6K pathways in RAW264.7 cells. RAW264.7 cells were treated with quercetin (10, 20 μM) for 2 h and then treated with RAP (100 μg/mL) for 30 min.

In our previous study, we proved that Akt downstream of the NFκB, MAPKs and IRF3 pathways is involved in macrophage activation induced by Astragalus polysaccharide RAP. In this study, we tested the effect of quercetin on the Akt pathway and its downstream mammalian target of rapamycin complex 1/S6 kinase (mTORC1/S6K) pathway in RAW264.7 cells induced by RAP. Western blot analysis suggests that quercetin treatment inhibits the Akt and mTORC1/S6K pathways (see Figure 7).

## 3. Discussions

This study shows that quercetin inhibits the production of inflammatory mediators , and that this inhibition is, at least partially, related to the inhibition of the phosphorylation of the Akt/mTORC1/S6K1, and TBK1 pathways and of MAPKs in RAP-induced macrophage cells. All the results suggest the conclusion that quercetin partially inhibits RAP activation of macrophages. These results suggest that quercetin-containing foods could interfere with, or reduce, the immunoregulatory effects of Astragalus because the polysaccharide RAP is one of its active components.

It has been reported that the stimulation of Toll-like receptor 4 facilitates the activation of two pathways: the MyD88-dependent, and the MyD88-independent. The MyD88-dependent pathway involves NF-κB and MAPKs activation, while the MyD88-independent pathway activates the TBK1/IRF3 pathway [19,20]. We have reported that the Astragalus polysaccharide RAP exhibits immunoenhancing effects in macrophages via TLR4 receptor and downstream kinases. We further showed that Akt can exert effects downstream of both MyD88-dependent and -independent pathways on macrophage activation induced by the polysaccharide RAP [21]. Other studies have also reported that Akt/mTOR signaling pathways downstream of TLR4 are involved in macrophage activation [22,23]. In this study, we first showed that quercetin can decrease inflammatory mediator production and gene expression level in RAP induced-RAW264.7 cells. We further found that quercetin can exert inhibitory effects on RAP-induced peritoneal macrophages. In other words, quercetin can inhibit the activation in both RAW264.7 cells and peritoneal macrophages. As for the mechanisms, we examined the signaling pathway downstream of the TLR4 receptor. Western blot analysis suggested that quercetin inhibited the phosphorylation of the Akt/mTORC1/S6K, MAPKs and TBK1 pathways, but had no effect on the NF-κB pathway in RAP induced RAW264.7 cells. More experiments should be conducted to clarify the exact underlying mechanisms, such as why quercetin inhibits only the Akt/mTORC1/S6K, MAPKs and TBK1 pathways and not the NF-κB pathway, and whether every pathway has the same function in the inhibitory effect of quercetin.

In our previous study, we reported that luteolin, a well-studied flavonoid, partially inhibits macrophages induced by RAP. In this case, the inhibition of the phosphorylation of NF-κB p65 and Akt, but not of MAPKs and IRF3 may be involved. All results demonstrate that quercetin and luteolin in foods can exert different effects on Astragalus polysaccharide-induced macrophage activation [24]. Quercetin and luteolin, which differ by one hydroxyl group, are known to suppress the lipopolysaccharide-induced production of TNF-α in vitro. One study reported that quercetin but not luteolin suppresses infection-induced lethal shock in mice [25]. The additional hydroxyl group in quercetin may be involved in the different effect.

The aim of the present study was to investigate the interaction between a flavonoid and an Astragalus polysaccharide. LPS-induced macrophage activation is a well-established model to evaluate anti-inflammatory effect. In LPS-induced macrophage activation, quercetin inhibits NK-κB, ERK1/2 and JNK activation [15], and suppresses the activation of ERK and p38 MAP kinases, and the NF-κB signal transduction pathways, but not JNK MAP kinase [11]. It has been reported that many polysaccharides, including those from Astragalus, could activate immune cells via the TLR4 receptor. Many of these natural polysaccharides have been reported to have LPS-like bioactivities. However, results demonstrate that how quercetin interacts with polysaccharides RAP-induced macrophages is different from the anti-inflammatory effects in LPS-induced macrophages. These results suggest that, while RAP shares some LPS immunomodulating effects with LPS, it is not identical to LPS. Different mechanisms appear to be involved in the macrophage activation induced by Astragalus polysaccharides compared with LPS.

Macrophages play an important role in immunity and immune responses. Macrophages may be the first line of tumor resistance, as they rapidly colonize and secrete cytokines that attack tumor cells, and they activate dendritic cells and natural killer cells [26]. Astragalus Polysaccharide is a traditional Chinese medicine to enhance innate immune functions and is becoming an important food additive. Astragalus polysaccharides have been shown to have diverse bioactivities, such as immunomodulatory [27,28] and anti-tumor properties [29,30]. It is well known the that there are many active flavonoids in common vegetables and fruits, that can exert anti-inflammatory and immunosuppressive effects. However, there has been little study of the interaction of the known active dietary flavonoids with these polysaccharides. Hence, the results presented here provide valuable information about the interaction between the foods which contain quercetin and one Astragalus polysaccharides. This study advances our understanding about the interaction between immunosuppressive flavonoids in daily foods and polysaccharides that exert immunoenhancing effects. On a practical level, it provides cautionary information for people who take quercetin-including foods with polysaccharides at the same time to consider. In addition to quercetin, other flavonoids have been reported to exert immunosuppressive activity [31,32]. Thus, more experiments should be conducted to investigate the interaction between flavonoids compounds and polysaccharides.

The functions of inflammatory factors are very complicated for health and can have the opposite effects in certain situation. The results of this study suggest that it is possible for quercetin to affect other immune cells. The interaction effect of quercetin with RAP could be complicated and needs to be tested in vivo. These points will be considered in the next studies. In this present study, we only speculate quercetin might be interfering with the immunoenhancing effect of RAP at this point.

## 4. Materials and Methods

### 4.1. Materials

LPS and Griess reagent (modified) were purchased from Sigma-Aldrich (St. Louis, MO, USA). Mouse TNF-α ELISA kit and IL-6 ELISA kit were purchased from eBioscience (San Diego, CA, USA). The antibody COX2, iNOS, phos-P65 (Ser536), P65, phos-TBK1, TBK1, phos-AKT (Ser473), AKT, phos-S6K (Thr389), S6K, phos–P38 (Thr180/Tyr182), P38, phos–JNK(Thr183/Tyr185), JNK, GAPDH (Cell Signaling, Danvers, MA, USA) and phos–ERK (Tyr204), ERK (Santa Cruz Biotechnologies, Santa Cruz, CA, USA), HRP-linked anti-mouse and anti-rabbit antibodies were obtained from Santa Cruz. SYBR Select Master Mix for RT-PCR amplification was purchased from Invitrogen Life Technologies (Carlsbad, CA, USA). Primers for TNF-α, IL-6, iNOS and GAPDH were from Invitrogen Life Technologies (Carlsbad, CA, USA).

### 4.2. RAP Preparation

The roots of Astragalus membranaceus were purchased from a herbal store in Hong Kong and identified by Dr. Chun-Feng Qiao. The voucher specimens are deposited at the Institute of Chinese Medicine, the Chinese University of Hong Kong, with voucher specimen number 2010–3268 [33]. The isolation and purification procedure were performed according to the previous study [33]. Briefly, the air-dried Radix Astragali was powdered and extracted twice with boiling water. The solution was filtered, combined, and concentrated. The solution was precipitated with absolute ethanol. The precipitate was resolved again in water and deproteined. Then the water solution was dialyzed. Finally, the retentate was lyophilized. The product was dissolved in distilled water again and separated with a Hiload 26/60 Superdex-200 column, eluted with water. Fractions were collected, dialyzed, and finally lyophilized to obtain RAP. The RAP used in this study was no endotoxin contaminated (see supplemental Figure S1).

### 4.3. RAW264.7 Cells Culture

RAW264.7 cells were cultured in DMEM supplemented with 10% heat-inactivated FBS, 100 U/mL penicillin, and 100 mg/mL streptomycin. Cells were cultured at 37 °C in a 5% CO_2_ incubator.

### 4.4. Peritoneal Macrophages

Thioglycolate-elicited mouse primary peritoneal macrophages were prepared from female C57BL/6J mice (6–8 weeks of age) as described previously [34]. After 2 h, non-adherent cells were removed, and the adherent cells were used as peritoneal macrophages.

### 4.5. NO Level

RAW264.7 cells were seeded in 96-well plates incubated for overnight, RAW264.7 cells were treated with quercetin (10, 20 μM) for 2 h, and then the cells were induced by RAP (100 μg/mL) or LPS (100 ng/mL) for 24 h. Each culture supernatant was mixed with griess reagent for 10 min at room temperature. The absorbance values were detected at 540 nm.

### 4.6. TNF-α and IL-6 Assay

The cells were treated with quercetin (10, 20 μM) for 2 h, and then exposed to RAP (100 μg/mL) or LPS (100 ng/mL) for 24 h. At the end of treatment, the medium was used for IL-6 and TNF-α determination. Cytokine TNF-α and IL-6 levels in the culture media were determined by using a colorimetric, commercial ELISA kit (eBioscience, San Diego, CA, USA).

### 4.7. Real-Time Quantitative PCR

To examine gene expression, RAW264.7 cells were treated with quercetin (10, 20 μM) for 2 h; then, RAP at 100 μg/mL was added, and the cells were incubated for another 24 h. The expression of iNOS, TNF-α and IL-6 genes was assessed by real-time RT-PCR using the SYBRs Select Master Mix (Life Technologies, Carlsbad, CA, USA) with ViiA™ 7 Real-Time PCR System. The following sequences for primers from 5′ to 3′ end were used: TNF-α: forward, 5′-ATGAGCACAGAAAGCATGATC-3′, TNF-α: reverse, 5′-TACAGGCTTGTCACTCGAATT-3′; IL-6: forward, 5′-GATGCTACCAAACTGGATATAATC-3′, IL-6: reverse, 5′-GGTCCTTAGCCACTCCTTCTGTG-3′; iNOS: forward, 5′-GCCGTGGCCAACATGCTACT-3′, iNOS:reverse, 5′-GGTCTTCCTGGGCTCGATCTG-3′; GAPDH: forward, 5′-TGACCACAGTCCATGCCATC-3′, GAPDH: reverse, 5′-GACGGACACATTGGGGGTAG-3′.

### 4.8. Western Blotting Analysis

RAW264.7 cells were treated with quercetin for 2 h; then, RAP at 100 μg/mL was added and the cells were incubated for 30 min or 24 h. After that, total protein was extracted, and 20 μg protein was loaded and separated by SDS–PAGE, and then transferred to the PVDF transfer membranes. The membranes were blocked in 3% BSA in Tris-buffer saline for 1 h and then incubated overnight with a primary antibody at 4 °C. The antibodies were used as indicated by the manufacturer’s instructions. After incubation with the peroxidase-conjugated goat anti-rabbit or anti-mouse secondary antibodies, the immunoreactivity was visualized using an ECL Kit (Amersham Pharmacia Biotech, Piscataway, NJ, USA).

### 4.9. Reporter Assays

Reporter plasmids were transiently transfected into cells. 24 h after transfection, RAW264.7 cells were pretreated for 2 h with quercetin and then stimulated with RAP for 12 h. Then luciferase reporter activity was assayed using the Dual Luciferase Assay System according to the manufacturer’s instructions.

### 4.10. Statistics

Data from the experiments are expressed as mean ± SD from a minimum of 3 independent experiments. A comparison between groups was carried out by one-way ANOVA or student *t* test, and a *p* value of less than 0.05 was considered significant.

## 5. Conclusions

The results show that quercetin inhibits inflammatory mediators production, and that this inhibition is, at least partially, related to the inhibition of the phosphorylation of the Akt/mTORC1/S6K1, and TBK1 pathways and of MAPKs in RAP-induced macrophage cells. This study demonstrates that quercetin-containing foods may interfere with the immune-enhancing effects of Astragalus polysaccharide RAP to a certain extent.

## Supplementary Materials

The following are available online: Figure S1.
