# Supplementary material for: Interference of Quercetin on Astragalus Polysaccharide-Induced Macrophage Activation
Source: Molecules. 2018 Jun 28;23(7):1563. doi: 10.3390/molecules23071563 (PMC6100010; doi:10.3390/molecules23071563)
Supplement: Supplementary file 1 [file molecules-23-01563-s001.pdf]

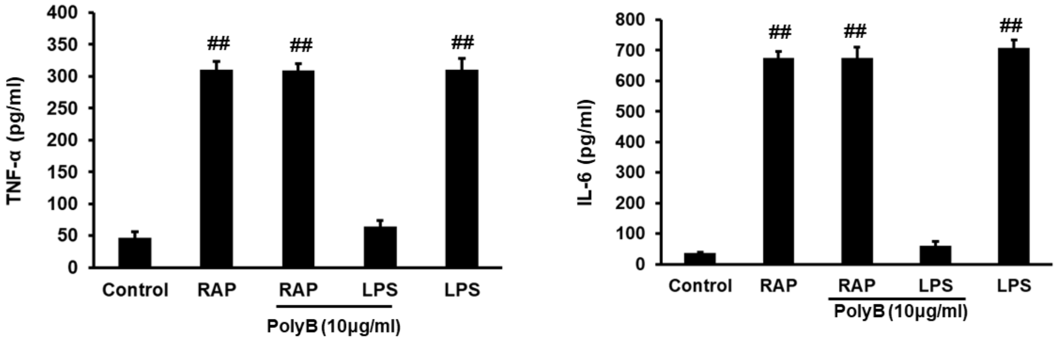

Supplementary Figure 1: RAW264.7 cells were treated with RAP (100μg/mL) or LPS (100ng/mL) with or without PolyB for 24h ($^{##}P < 0.01$ compared with control group).
